# Implementation of an innovative web base support system (Psynary) and Nurse Practitioner led service to support optimisation of treatment for depression (OptiMA2)

**DOI:** 10.1192/j.eurpsy.2022.1452

**Published:** 2022-09-01

**Authors:** R. Kabaila, R. Tranter, P. Latimer, R. Oakeshott

**Affiliations:** NSW Health, Community Mental Health, Port Macquarie, Australia

**Keywords:** Depression, Research, nurse practitioner, innovation

## Abstract

**Introduction:**

There is overwhelming evidence to show that achieving full remission in depression is important — especially in reducing the indirect costs of depression. Evidence further demonstrates that in primary care, clinicians are not optimising treatments for depression in a timely way —resulting in them not being able to achieve early remission for their clients experiencing depression. Presently, secondary care is unable to provide specialist input for this client cohort.

**Objectives:**

This project is implementing a model which extends specialist care to primary care. This project assists GP’s through optimising treatments for clients presenting with moderate to severe depression This model uses nurse practitioner led care, with ‘Psynary’, an online system which optimises treatments for moderate to severe depression.

**Methods:**

Mixed methods pilot service implementation study, utilising: literature review of published service implementation models; service data gap analysis; qualitative interviews and focus group methodology.

**Results:**

GP and client focus group outcomes, as well as client remission rates in the OptiMA2 trial demonstrate that this healthcare pathway is effective.

**Conclusions:**

The OptiMA2 trial focused on the qualitative analysis of the co-design process to implement the initial care pathway.The OptiMA3 trial will examine the cumulative clinical outcomes to consider if increased rates of remission are achieved and identify potential predictive factors. The long term goal for the system is to support the development of community based care-extender models, including specialist nurses, pharmacists and GPs, to extend specialist mental health expertise to larger primary care populations where the greatest burden of mental illness occurs.

**Disclosure:**

No significant relationships.

